# Demographic and Clinical Characteristics of Early‐Onset Colorectal Cancer in Sweden and Finland: A Multicentre Retrospective Cohort Study Over Three Decades

**DOI:** 10.1002/jso.70246

**Published:** 2026-04-02

**Authors:** Melina Charalambidi, Tanja Hukkinen, Tuomas Kaprio, Sofia Edin, Mats Hjortborg, Cecilia Williams, Jaana Hagström, Caj Haglund, Richard Palmqvist, Karin Strigård, Camilla Böckelman, Ioannis Gkekas

**Affiliations:** ^1^ Department of Diagnostics and Intervention Surgery Umeå University Umeå Sweden; ^2^ Department of Gastrointestinal Surgery University of Helsinki and Helsinki University Hospital Helsinki Finland; ^3^ Department of Medical Biosciences Pathology Umeå University Umeå Sweden; ^4^ Department of Protein Science, SciLifeLab KTH Royal Institute of Technology Solna Sweden; ^5^ Department of Medicine Huddinge Karolinska Institute Huddinge Sweden; ^6^ Department of Oral Pathology and Radiology University of Turku Turku Finland

**Keywords:** changes over time, early‐onset colorectal cancer, Finland, mismatch repair deficiency, Sweden, tumour characteristics

## Abstract

Colorectal cancer is the third most common cancer worldwide, and the proportion of individuals diagnosed under the age of 50 years, referred to as early‐onset colorectal cancer (EOCRC), is increasing. The aim of this study was to evaluate how the demographic and clinical features of EOCR in northern Sweden and Finland have changed over time. All patient data were extracted from local hospital surgical department databases between 1995 and 2022. Two CRC cohorts, Study Cohort I (1995–2005) 1237 patients and Study Cohort II (2006–2022) 4526 patients, were compared for age, sex, disease stage, tumour grade, tumour location, and mismatch repair status. EOCRC patients comprised 7% of all CRCs in Study Cohort I and 4% in Study Cohort II. The mean ages were 42 and 43 years respectively, and 55% of patients were female. The vast part of EOCRC tumours were left‐sided stage III–IV cancers. Most tumours (*n* = 204, 73%) were low grade, and 10% showed mismatch repair deficiency. No significant differences in demographic or tumour characteristics were seen over time. EOCRC in northern Sweden and Finland is characterised by advanced‐stage, low tumour grade, a slight female predominance, and stable clinical and pathological features. These findings partly contrast with reports on EOCRC from other high‐income countries, highlighting the need for further research on advanced molecular characteristics and potential gender differences in incidence and survival of this population.

## Summary

- EOCRC in northern Scandinavia shows stable clinical features over time.
- No changes in demographic or tumour characteristics were observed.
- Most EOCRC tumours are left‐sided, low grade, and diagnosed at advanced stages.

**Abbreviations**
APC: Adenomatous Polyposis Coli
CIMP: CpG Island Methylator Phenotype
CIN: Chromosomal Instability
CRC: Colorectal Cancer
Dmmr: Deficient Mismatch Repair
EOCRC: Early‐Onset Colorectal Cancer
LOCRC: Late‐Onset Colorectal Cancer
MACS: Microsatellite‐ and Chromosomal‐Stable Tumours
MMR: Mismatch Repair
MSI: Microsatellite Instability
MSS: Microsatellite Stability
pMMR: Proficient Mismatch Repair
TNM: Tumour‐Node‐Metastasis

## 1 Introduction

Colorectal cancer (CRC) is the third most common cancer globally, accounting for close to 2 million new cases in 2023, and it is the second leading cause of cancer‐related mortality [1, 2]. Although the risk for CRC increases with age, the incidence of CRC in the population over 50, referred to as late‐onset colorectal cancer (LOCRC), has decreased by approximately 3.3% each year between 2000 and 2016, probably due to increased screening [2, 3]. Conversely, the incidence of early‐onset colorectal cancer (EOCRC), defined as diagnosis before 50, increased approximately 2% each year over the same period [1, 2, 3, 4]. Lifestyle factors may play an important role in colorectal carcinogenesis as increases in EOCRC incidence are largely confined to high‐income countries, including Sweden [1, 2, 5]. Dietary factors such as high consumption of red meat, processed foods, and poor intake of dietary fibre (the “Western diet”) have been suggested as modifiable risk factors [1, 4, 6, 7]. In North America, EOCRC is expected to account for approximately 11% of all colon cancer cases by 2030, and close to 23% of all rectal cancers [4].

EOCRC tends to be diagnosed at a more advanced stage, possibly due to the fact that national screening programmes do not include younger patients, as well as misinterpretation of symptoms in younger patients by both patients and clinicians [4, 8]. LOCRC usually presents as a change in bowel habit, whereas abdominal discomfort or pain are most common in EOCRC [9, 10]. Apart from being diagnosed at a more advanced stage, EOCRC also tends to metastasise easier, both synchronously and metachronously [8, 11, 12]. Nevertheless, EOCRC has a better overall survival than LOCRC, probably due to younger age, more aggressive treatment options, and less comorbidity [8]. Differences in survival could also be related to differences in molecular characteristics, however, there is no consensus on the molecular characteristics of metastatic EOCRC [4, 8].

There are three main molecular profiles used to classify CRC: chromosomal instability (CIN), microsatellite instability (MSI), and the CpG island methylator phenotype (CIMP). The CIN pathway occurs predominantly in sporadic EOCRC, and accounts for approximately 85% of all cases, compared to about 70% in LOCRC [10]. The CIN pathway more often leads to a left‐sided tumour [4, 9], is characterised by loss‐of‐function mutations in the adenomatous polyposis coli (APC) gene [9, 10], and is associated with microsatellite stability (MSS) [6]. Furthermore, the MSI pathway is associated with a deficient mismatch repair system (dMMR), which can arise from hypermethylation of the promotor of *MLH1*, somatic mutations or from genetic autosomal dominant germline mutations (such as the Lynch syndrome) in the MMR‐genes (*MLH1, MSH2, MSH6* and *PMS2*) [9, 10]. Studies on the association between EOCRC and the MSI pathway have yielded conflicting results. Nevertheless, EOCRC MSI tumours tend to be associated with inactivation of *MSH2* to a larger degree than LOCRC tumours that are associated with inactivation of *MLH1* [8]. Furthermore, dMMR appears to occur more frequently in metastatic EOCRC than in LOCRC [4, 8]. However, MSI tumours, both in EOCRC and CRC in general, have a better overall prognosis than MSS tumours [8, 9]. Finally, the CIMP pathway is reported to be more common in EOCRC than in LOCRC [13].

In Sweden, the prevalence of obesity among individuals under the age of 40 has increased fourfold since the 1980s [14]. This rise in obesity is concerning since it is a known risk factor for CRC and has made a substantial contribution to the approximately 6,000 new cases each year in Sweden. Notably, 4% of colon cancer cases and 5% of rectal cancer cases are now classified as EOCRC [15]. Likewise, in Finland, approximately 4,000 new cases of CRC are diagnosed each year with around 5% of these being classified as EOCRC [16]. A Swedish national CRC screening programme has been ongoing since 2023 and includes the population between 60 and 74‐years‐of‐age. The screening procedure includes faecal immunochemical analysis to detect human blood, followed by colonoscopy in positive cases [17]. A similar screening programme in Finland began in 2022, including all citizens between the ages of 60 and 68, and by 2031, is planned to include all citizens between 56 and 74 [18]. Before full national rollout, Sweden already had colorectal cancer screening in place. Regional programs began in 2008–2009, and national recommendations were issued in 2014 for biennial stool testing in adults aged 60–74. A national program started implementation in 2021, and all regions had initiated screening by September 2022 [19]. In Finland, CRC screening was conducted earlier through a nationwide program (2004–2016) for ages 60–69. After suspension, a FIT‐based pilot restarted screening in 2019 in selected municipalities. These pilots led to a new national FIT screening program launched in 2022, initially for ages 60–70 [18].

The primary aim of this study was to investigate the demographic and clinical features of EOCRC patients in Sweden and Finland, and to explore any changes over the period of the study. Our hypothesis was that the disease pattern has changed over that time.

## 2 Material and Methods

### 2.1 Study Population

CRC patients treated at Umeå University Hospital (Västerbotten County, Sweden) between 1995 and 2005, and at Helsinki University Hospital (Helsinki, Finland) between 1998 and 2005, were compared to patients treated at the same institutions during the periods 2006–2022 and 2006–2009 respectively. All patient data were extracted from local hospital surgical department databases and patient files. In the preliminary search, the ICD codes “C18.1‐9 and C20” were used for identification of CRC patients. Cases were divided between two time periods: 1995–2005 (1237 CRC patients: Study Cohort I) and 2006–2022 (4526 CRC patients: Study Cohort II). Both hospitals serve as the primary referral centres for their respective regions, and no significant patient migration to other institutions or regions was observed. These cases thus represent baseline populations within each region and include all EOCRC cases during each period that fulfilled the inclusion criteria. Cohort I, a previously studied population, was well‐characterised, whereas Cohort II was a new complementary cohort specifically chosen for this study. This difference motivated dichotomisation of the population around the years 2005/2006, a timepoint that also coincides with the period when early‐onset CRC began to gain increasing attention in Scandinavia [20, 21]. For Finland, the inclusion period ends in 2009 because complete and standardized EOCRC records from Helsinki University Hospital were only available up to that year.

### 2.2 Inclusion and Exclusion Criteria

The inclusion criteria were as follows: CRC patients aged 18–49 at the time of diagnosis, regardless of subsequent treatment; histologically confirmed CRC type adenocarcinoma; and available clinical and demographic data. Although patients were included regardless of treatment, pathological TNM staging was available for the majority because most EOCRC patients underwent surgical resection as part of standard clinical management at both centers. Tumours arising from the appendix and neuroendocrine tumours were excluded. Cases of recurrence and multiple synchronous tumours were excluded from the study, because these cases cannot be assigned a single set of clinicopathological characteristics, and their inclusion would introduce misclassification bias.

### 2.3 Determination of Clinical and Demographic Characteristics

Patient data included age, sex, grade of the tumour where high grade = undifferentiated and poorly differentiated, and low grade = moderately and highly differentiated. Cancer stage was according to the classification of the 8:th edition of the American Joint Committee on Cancer (AJCC)(All TNM stages represent pathological staging based on postoperative histopathological assessment, as clinical staging was not used for the cohort comparisons); tumour (T), regional lymph nodes (N), and distant metastasis (M) (TNM) [22]. Tumour localisation was categorised as right‐ or left‐sided colon divided about the splenic flexure, and rectal tumours including the rectosigmoid junction. MMR status was determined by immunohistochemical analysis of four DNA MMR proteins (MLH1, MSH2, MSH6 and PMS2) and classified as either proficient MMR (pMMR) or deficient (dMMR). Loss of nuclear expression of at least one of the four proteins indicates dMMR and presence of all proteins indicates pMMR. Before 2020, MMR status was not a routine part of the CRC diagnostic procedure in Sweden. Due to this and the retrospective study design, MMR status was not available for all Swedish participants, whereas all Finnish patients were analysed for MMR status.

### 2.4 Statistical Analysis

All statistical analyses were conducted in IBM SPSS Statistics 28 for Mac (IBM SPSS Statistics, version 28; IBM, Chicago, IL, USA). The independent t‐test was used for continuous variables, and the χ^2^ test used for categorical variables. In case of an expected cell count under five, Fisher's exact test was used. Crosstabulation analyses were used to evaluate differences in outcome between Study Cohorts I and II. A scatter plot with a linear regression analysis was used to evaluate the Swedish EOCRC proportion relative to all CRC cases in the primary selection group for each year and for each cohort respectively (1995–2005; 2006–2022). A *p*‐value less than 0.05 was considered significant.

### 2.5 Ethical Consideration

Ethics approval (Nr. 2024‐00110‐01) was given by the Swedish Ethics Review Authority prior to study initiation. In Finland, permission was granted by the Finnish Medicines Agency (permit for research conducted on human samples: Dnro FIMEA/2021/006901), the Hospital district of Helsinki and Uusimaa (Nr. HUS/23/2024), and the Ethics Committee of Medicine of the Helsinki University Hospital (Nr. HUS/1223/2021).

## 3 Results

### 3.1 Demographic and Clinical Features of the Study Population

Overall demographic and clinical features of the study populations are presented in Table 1, showing a total of 281 EOCRC patients diagnosed between 1995 and 2022. Of the study population, 59% (*n* = 167) were from Sweden and 41% (*n* = 114) were from Finland. The mean age at diagnosis was 43 (interquartile range (IQR) 41–48) and 55% (*n* = 155) were female. The tumour was located in the rectum in 43% (*n* = 121) and in the colon in 57% (*n* = 160), with 38% (*n* = 61) of the latter being right‐sided and 62% (*n* = 99) left‐sided. Regarding TNM stage, 14% (*n* = 38) presented with stage I, 23% (*n* = 66) with stage II, 36% (*n* = 101) with stage III, and 21% (*n* = 58) with a stage IV tumour. Furthermore, 73% (*n* = 204) of the study population presented with a low‐grade, and 19% (*n* = 54) with a high‐grade tumour. MMR status revealed 10% (*n* = 28) to be dMMR and 49% (*n* = 139) to be pMMR, with 41% (*n* = 114) of cases lacking MMR data.

**Table 1:** Demographic and clinical features of all EOCRC cases diagnosed 1995 to 2022 and separately for the two study periods 1995–2005 and 2006–2022.

	Study population 1995–2022	Study Cohort I (1995–2005)	Study Cohort II(2005–2022)	*p* value
EOCRC patients, *No* (%)	281 (5)	79 (6)	202 (4)	0.903
Median age	42.96	42.47	43.45	
Range	21–50	26–50	21–50	
IQR	41–48	41.6–48.0	41–47	
Sex, *No* (%)				
Male	126 (45)	37 (47)	88 (44)	0.901
Female	155(55)	42 (53)	114 (56)	
Location, *No* (%)				
Colon	160 (57)	44 (56)	116 (54)	0.506
Rectum	121 (43)	35 (44)	86 (46)	
Location colon, *No* (%)				
Right Colon	61(38)	20 (45)	41 (35)	0.148
Left Colon	99 (62)	24 (55)	75 (65)	
TNM stage, *No* (%)				
I	38 (14)	13 (16)	25 (14)	0.908
II	66 (23)	19 (26)	47 (26)	
III	101 (36)	31 (39)	70 (38)	
IV	58 (21)	16 (20)	42 (23)	
Missing	18 (6)			
TNM stage, *No* (%)				
I + II	104 (37)	32 (41)	72 (39)	0.899
III + IV	159 (57)	47 (59)	112 (61)	
Missing	18 (6)			
Tumour, *No* (%)				
Low grade	204 (73)	49 (62)	155 (81)	0.247
High grade	54 (19)	18 (23)	36 (19)	
Missing	23 (8)			
MMR status, *No* (%)				
dMMR	28 (10)	9 [17]	19 (17)	0.980
pMMR	139 (49)	45 (83)	94 (83)	
Missing	114 (41)			

*Note:* No = number, (%) = percent.

Missing cases: TNM stage (*n* = 18), tumour grade (*n* = 23) and MMR status (*n* = 114).

Abbreviations: dMMR, Deficient mismatch repair; IQR, Interquartile range; MMR, mismatch repair; pMMR, Proficient mismatch repair; TNM, Tumour‐Node‐Metastasis.

### 3.2 No Change in Distribution of Age, Sex, or EOCRC Proportion Was Seen Over Time

**Figure 1:**
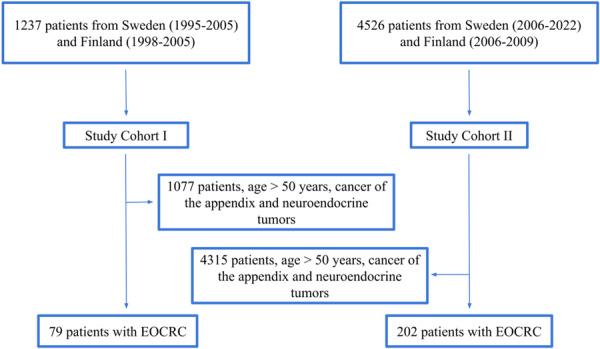
Flow chart of patient inclusion and exclusion. EOCRC = early‐onset colorectal cancer.

**Figure 2:**
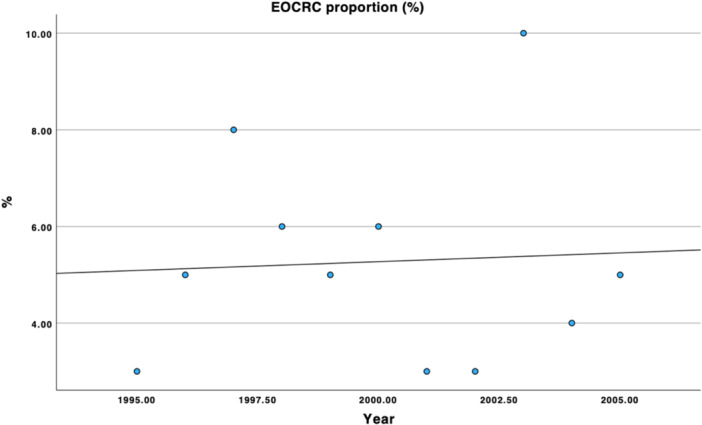
EOCRC proportions relative to all patients in the Swedish CRC cohort between 1995 and 2005. X‐axis: year, Y‐axis: EOCRC proportion in per cent (%). The fitted regression line (y = 0.036x‐67.4) indicates no significant change over time (*p* = 0.873). EOCRC = early‐onset colorectal cancer.

**Figure 3:**
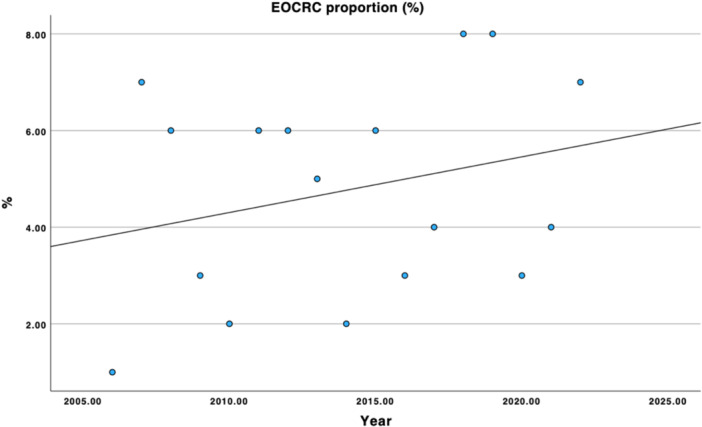
EOCRC proportions relative to all patients in the Swedish cohort of CRC cases between 2006 and 2022. X‐axis: year, Y‐axis: EOCRC proportion in per cent (%). The fitted regression line (y = 227 + 0.115x) indicates no significant change over time (*p* = 0.304). EOCRC = early‐onset colorectal cancer.

Altogether 79 patients in Study Cohort I and 202 patients in Study Cohort II were included in the analyses (Table 1), and a flow chart of the patient inclusion/exclusion process is shown in Figure 1. The median age of the EOCRC patients was 42 years (IQR 41.6–48.0) in Study Cohort I and 43 years (IQR 41–47) in Study Cohort II. Of the primarily selected CRC populations, EOCRC represented 7% of all CRC patients diagnosed between 1995 and 2005 compared to 4% of those diagnosed between 2006 and 2022 respectively. In the Swedish Study Cohorts, the proportion of EOCRC relative to all CRC cases did not show a significantly rising trend using a linear regression analysis (*p* = 0.873, Figure 2, *p* = 0.304, Figure 3). Still, we observed a concerning, albeit not statistically significant annual increase for both Swedish cohorts. A corresponding analysis of the Finnish cohorts was not deemed appropriate due to the substantially smaller sample size and a more limited time frame, which limited the ability to draw meaningful conclusions about temporal trends. The female proportion similar in both Study cohorts (53% and 56%). There was no significant sex‐ or age‐related differences over time (*p* = 0.901 and *p* = 0.903, Table 1). No significant difference in MMR status was observed between cohorts (*p* = 0.980). MMR status was determined in 77% (*n* = 54).

### 3.3 There Was a Trend Towards an Increase in Left‐Sided Tumours Over Time

In Study Cohort I, a colon tumour was observed in 56% (*n* = 44) and a rectal tumour in 44% (*n* = 35) of the patients. Similarly, in Study Cohort II, 54% (*n* = 116) presented with a colon tumour and 46% (*n* = 86) with a rectal tumour. Of the colon tumours, 55% were left‐sided in Study Cohort I compared to 65% in Study Cohort II. There was no significant difference in tumour location over time between the cohorts (*p* = 0.506), however, a non‐significant increase in left‐sided tumours was observed (55% vs. 65%, *p* = 0.148).

### 3.4 Most Tumours Were Stage III and IV at Diagnosis, With No Difference Between Cohorts

No significant differences were observed between the two cohorts regarding individual TNM stages, nor when grouped into combined Stages I–II and Stages III–IV respectively (*p* = 0.908 and *p* = 0.899). TNM staging in Study Cohort I revealed 16% (*n* = 13) with Stage I, 24% (*n* = 19) with Stage II, 39% (*n* = 31) with Stage III, and 20% (*n* = 16) with Stage IV at diagnosis. In Study Cohort II, TNM staging was available for 91% (*n* = 184) of the population, with 14% (*n* = 25) Stage I, 26% (*n* = 47) Stage II, 38% (*n* = 70) Stage III, and 23% (*n* = 42) Stage IV at diagnosis.

## 4 Discussion

In this multicentre study, which included two large cohorts from medical centres in Umeå, Sweden and Helsinki, Finland, no statistically significant demographic or clinical differences were observed among EOCRC patients over a period spanning three decades. In the present study, the EOCRC patients tended to present with stage III or IV disease, along with a higher proportion of rectal and left‐sided colon tumours. In addition, most tumours were pMMR. Our findings concur with other reports indicating that EOCRC pMMR tumours are more likely to be diagnosed at an advanced stage and are predominantly located in the left colon [23]. Furthermore, EOCRC is associated with high grade tumours with a higher prevalence of mucinous and poorly differentiated cells [10, 24, 25], as well as more aggressive disease with high metastatic potential [8, 26, 27]. EOCRC generally presents with higher TNM stage disease, predominantly located in the distal colon and rectum [10]. However, our results indicate that these Scandinavian cohorts present with a low‐grade tumour, typically associated with a less aggressive disease profile, indicating an area for future studies. Although, because Sweden and Finland have historically lacked CRC screening for individuals under 50—unlike countries such as the United States where screening now begins at age 45—our findings primarily reflect symptomatic EOCRC presentations rather than true incidence, which may limit direct comparability with screened populations. Given these demographic shifts and changes in screening practices, we cannot determine whether the true incidence of EOCRC has changed in the population over time.

It should be noted that there was no evidence to support a significantly increasing proportion of EOCRC related to all CRC over time. This finding contradicts the increasing incidence of EOCRC seen worldwide and especially in the North American population [7]. However, the lower proportion of cases with EOCRC seen in this study may reflect the increased implementation of CRC screening among older individuals in Sweden over recent decades, despite it not being generally recommended until 2023 [17]. Since screening initially leads to a higher number of tumours being detected in the older population, the proportion of EOCRC cases falls as a result. In Sweden, screening activity increased prior to national guideline implementation, with all 21 regions initiating organized CRC screening between April 2021 and September 2022, expanding invitations to roughly 1.65 million individuals aged 60–74. Furthermore, the median age of the Swedish population has risen since 1998, which affects the relative proportions of older versus younger individuals [28]. This shift in age distribution likely contributed to the observed decrease in the proportion of EOCRC cases within the total CRC populations between 1995 and 2005 and 2006–2022, although the difference was not statistically significant. Also, we did not observe a statistically significant increase in the Swedish EOCRC proportion on an annual basis between 1995 and 2005, and 2006 and 2022 respectively, likely due to the same reasons mentioned above. Importantly, as the Swedish data span a longer period than corresponding data from the other cohort, they provide a valuable basis for assessing long‐term trends in EOCRC within the context of a stable population. In addition, the data available for the Finnish population were more limited in scope, which influenced our decision to focus this particular analysis on the Swedish cohort.

In 2019, a global survey of the incidence of EOCRC over two decades, showed the incidence rate among men to be increasing by approximately 2% compared to women [29]. In the present study, 55% of the total EOCRC population were women. Research suggests that the overall survival time for men is shorter than for women, especially among EOCRC patients [30]. Higher oestrogen levels among premenopausal women could explain the lower CRC incidence and better survival rate seen among young females [31, 32, 33]. Overall survival was not addressed in this study, but the fact that 55% of EOCRC in the Swedish and Finnish cohorts were women seems to contradict the findings of previous studies, and this again warrants further investigation.

Approximately half of MSS tumours in an EOCRC population are reported to be CIN‐negative and classified as microsatellite and chromosomal stable tumours (MACS) [34]. This was not investigated in the present study. As with MSS tumours, MACS primarily affects the distal colon and is associated with a more aggressive disease profile [23, 34]. Moreover, Lynch syndrome is more strongly associated with EOCRC (approximately 20%) than with LOCRC (2‐3%) [8]. These EOCRC tumours tend to be right‐sided and have a higher metastatic potential compared to LOCRC. Overall, though, they have a better prognosis [8, 10]. Despite the higher prevalence of hereditary syndromes among EOCRC, these are not considered responsible for the recent increase in EOCRC [24]. Since most young individuals are not aware of their hereditary predisposition, population‐based figures are difficult to obtain [35]. This should also be explored in future studies, and our research group has already started data collection to test potential differences in the Scandinavian population.

Strengths of the present study include the fact that all data were collected using a standardised protocol in both Finland and Sweden, thereby minimising the risk for subjective interpretation and enhancing internal validity. Moreover, the inclusion of patients from two different countries represents a particular strength, as it enabled the construction of a larger, more heterogeneous cohort while still maintaining cultural and healthcare system compatibility. This is especially important given that this is the first study to map the characteristics of EOCRC in northern Scandinavia over time, whereas most existing data have been derived from North American populations [2, 3]. Even in those settings, however, differences in EOCRC, including baseline characteristics, have yet to be explored comprehensively.

Limitations of this study are that retrospective cohort studies are prone to error, particularly in the collection of patient data, which may vary between countries. Due to the retrospective nature of this study, MMR status was not available for all Swedish participants, limiting the possibility of a more robust analysis. Because pathological TNM staging was only available for patients who underwent surgery, individuals with unresectable or palliative disease are likely underrepresented, which may introduce a selection bias toward operable cases. There were some discrepancies between the Swedish and Finnish populations during the time periods available, and these are seen as a limitation of the study. Finally, the high proportion of missing MMR data (41%), largely due to the absence of routine MMR testing in earlier years, reduces the power of MMR‐specific analyses but is unlikely to bias comparisons between cohorts since the missingness was non‐differential.

This study concludes that the nature of EOCRC over time in northern Sweden and Finland is characterised by high disease stage and low tumour grade at primary diagnosis. The results, however, do not show any statistically significant changes in clinical and demographic characteristics over time, nor was there any significant increase in aggressivity between the two time periods. These findings suggest the need for further research on advanced molecular characteristics and possible gender differences in relation to incidence and overall survival of EOCRC.

## Funding

This project was supported by grants from the Cancer Foundation in Northern Sweden and the Finska Läkaresällskapet (Camilla Böckelman, Caj Haglund, and Tanja Hukkinen), the Sigrid Jusélius Foundation (Caj Haglund), Medicinska understödsföreningen Liv och Hälsa (Camilla Böckelman, Caj Haglund and Tuomas Kaprio).

## Conflicts of Interest

The authors declare no conflicts of interest.

## 1 Data Availability Statement

The data that support the findings of this study are available on request from the corresponding author. The data are not publicly available due to privacy or ethical restrictions.

## Synopsis

This multicentre cohort study examines temporal trends in early‐onset colorectal cancer in Sweden and Finland over 30 years and finds stable demographic and clinicopathological characteristics over time. Despite global increases in EOCRC, Scandinavian patients predominantly presented with left‐sided, low‐grade, advanced‐stage tumors.
